# QKI-7 Regulates Expression of Interferon-Related Genes in Human Astrocyte Glioma Cells

**DOI:** 10.1371/journal.pone.0013079

**Published:** 2010-09-29

**Authors:** Lin Jiang, Peter Saetre, Katarzyna J. Radomska, Elena Jazin, Eva Lindholm Carlström

**Affiliations:** 1 Department of Development and Genetics, Uppsala University, Uppsala, Sweden; 2 Department of Clinical Neuroscience, HUBIN Project, Karolinska Institute and Hospital, Stockholm, Sweden; University of North Dakota, United States of America

## Abstract

**Background:**

The human QKI gene, called *quaking homolog*, *KH domain RNA binding (mouse)*, is a candidate gene for schizophrenia encoding an RNA-binding protein. This gene was shown to be essential for myelination in oligodendrocytes. QKI is also highly expressed in astrocytes, but its function in these cells is not known.

**Methods/Principal Findings:**

We studied the effect of small interference RNA (siRNA)-mediated QKI depletion on global gene expression in human astrocyte glioma cells. Microarray measurements were confirmed with real-time quantitative polymerase chain reaction (qPCR). The presence of QKI binding sites (QRE) was assessed by a bioinformatic approach. Viability and cell morphology were also studied. The most significant alteration after QKI silencing was the decreased expression of genes involved in interferon (IFN) induction (P = 6.3E-10), including IFIT1, IFIT2, MX1, MX2, G1P2, G1P3, GBP1 and IFIH1. All eight genes were down-regulated after silencing of the splice variant QKI-7, but were not affected by QKI-5 silencing. Interestingly, four of them were up-regulated after treatment with the antipsychotic agent haloperidol that also resulted in increased QKI-7 mRNA levels.

**Conclusions/Significance:**

The coordinated expression of QKI-7 splice variant and IFN-related genes supports the idea that this particular splice variant has specific functions in astrocytes. Furthermore, a role of QKI-7 as a regulator of an inflammatory gene pathway in astrocytes is suggested. This hypothesis is well in line with growing experimental evidence on the role of inflammatory components in schizophrenia.

## Introduction

QKI protein is a member of the STAR (Signal Transduction and Activation of RNA) family [1]. This family of proteins contains an RNA binding domain called KH [2]. In particular, the KH domain of QKI is extremely conserved among different species, and binds selectively to cellular messenger RNAs [3], many of which contain a specific QKI response element (QRE) [4]. In other words, QKI post-transcriptionally regulates expression of several RNAs by directly binding to them at QRE sites. There are three major splice variants called QKI-5, QKI-6 and QKI-7 that encode different protein isoforms.

QKI has been proposed as a candidate gene for schizophrenia after several lines of evidence including linkage analysis [5], [6] and mRNA expression studies [6], [7], [8]. Although decreased QKI expression in the brain of schizophrenia patients has been shown by several groups, the nature of the cells with reduced expression, and the physiological effect in the brain of patients is not known. Previous research efforts concentrated on the study of QKI function in oligodendrocytes because multiple lines of evidence implicate this cell type in schizophrenia [9], [10], [11], [12]. Indeed, the QKI protein has been shown not only to be necessary, but also sufficient to promote differentiation of oligodendrocytes into myelinating cells in rats [13]. However, all three QKI proteins are highly expressed in all glial cells in mice, including not only oligodendrocytes but also astrocytes [14], which are the most abundant glial cells in the brain [15]. Moreover, a role of astrocytes in schizohrenia has been already proposed based on several lines of evidence [16], [17], [18], [19], [20], [21], [22].

In order to study the role of QKI in astrocytes, we used small interference RNA (siRNA) designed to suppress the expression of specific QKI splice variants in human astrocyte glioma cells, and studied the effect of silencing on global gene expression.

## Results

### Silencing the expression of QKI-tot, QKI-5 and QKI-7 in human glial cell lines

**Figure 1:**
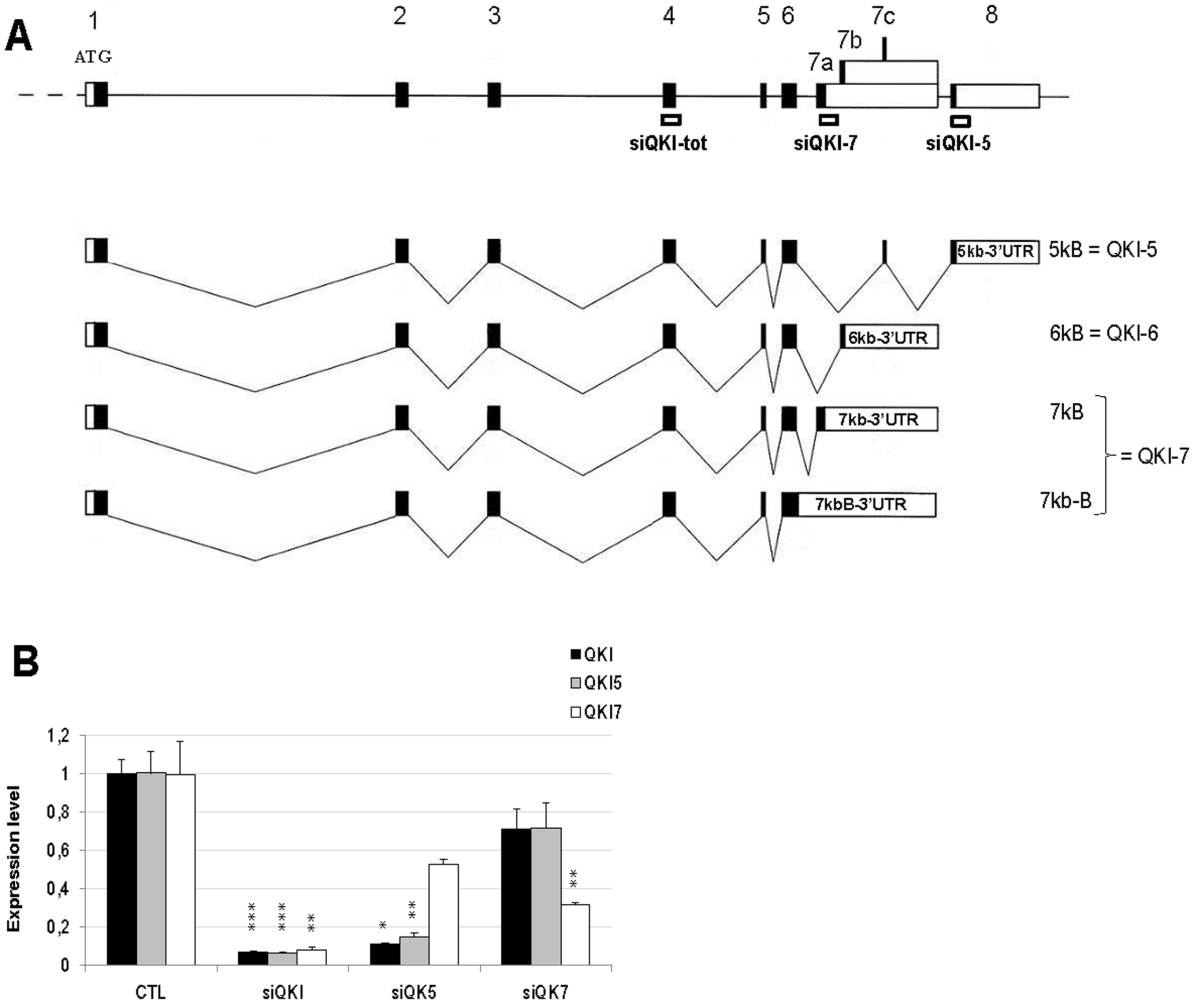
Small interference RNA (siRNA)-mediated depletion of QKI splice variants in astrocytoma (U343) cell lines. A. Exon-intron structure of the quaking gene and the regions targeted by small interfering RNA. siQKI-tot was designed to suppress all QKI splice variants (QKI-tot), siQKI-5 silences only splice variant 5 (QKI-5), and siQKI-7 silences splice variants 7 and 7b (QKI-7). The sequences included in each siRNA cocktail are listed in Table 2. B. Percentage of remaining messenger RNA expression levels for QKI-tot, QKI-5 and QKI-7, are shown after silencing with siQKI-tot, siQKI-5 or siQKI-7. Asterisk (*) indicates significant deviation in the mRNA levels compared with the untreated (CTL) groups (p-value <0.05, <0.01, and <0.001 for one, two, and three asterisks, respectively). Mean and standard errors are given based on three biological replicates.

Astrocyte glioma cells were treated with three different pools of siRNA designed to silence all QKI splice variants (QKI-tot), or only splice variant QKI-5 or QKI-7 (Fig. 1A). QKI-6 sequence does not contain splice-variant specific fragments that could be used for siRNA design (Fig. 1A). The remaining expression levels after silencing were measured by real-time RT-PCR (Fig. 1B). The expression of all splice variants was significantly reduced in the cells treated with the siRNA that targets all transcripts, with less than 10% of QKI expression levels remaining (Fig. 1B). The siRNA for the splice variants 5 and 7 (siQKI-5 and siQKI-7) reduced the corresponding splice variant. In addition, silencing of QKI-5 also resulted in decreased expression of the other splice variants (Fig. 1B), suggesting that QKI-5, which is located in the nucleus, may affect mRNA levels of the other splice variants, that are predominantly located in the cytoplasm [14]. In summary, we were able to efficiently silence QKI in astrocyte glioma cells.

### Global analysis of expression differences in QKI-Silenced Glial Cell Lines

QKI is known to regulate RNA expression levels of multiple genes and we reasoned that the silencing experiments should modify mRNA expression patterns in the cells. To study this, we used microarray analysis as described in the Methods. In total, 143 annotated genes were differentially expressed as shown in Supplementary Table S1 (Table S1). The genes with altered expression generated three gene groups, depending on whether the cells were silenced with siQKI-tot, siQKI-5 or siQKI-7. Detailed information such as gene names, fold changes, and expression in patients (previously reported by others), are summarized in Table S1. As a control, we repeated all siRNA experiments in a human oligodendroglial cell line named HOG, previously established by others [23].

### GO and Ingenuity Analysis

**Figure 2:**
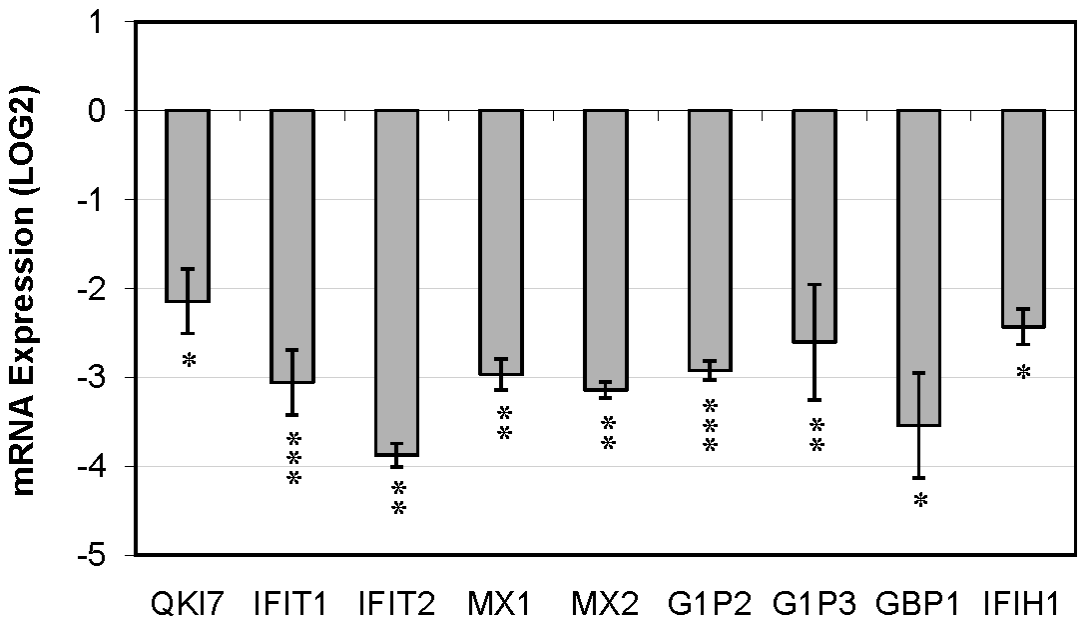
Real-time qPCR experiments confirm decreased mRNA Expression of Interferon-inducible genes in QKI-7 silenced U343 cells. Messenger RNA expression levels of IFIT1, IFIT2, MX1, MX2, G1P2, G1P3, GBP1 and IFIH1, relative to reference gene expression (ACTB and GAPDH) in QKI-7 silenced cells were measured by real-time RT-PCR to confirm the results of microarray experiments. As a control for the efficiency of silencing, the levels of QKI-7 were also measured. The level in untreated cells is defined as zero. Asterisk (*) indicates significant deviation in the mRNA levels compared with the untreated cells (p-value <0.05, <0.01, and <0.001 for one, two, and three asterisks, respectively). Mean and standard errors are given based on three biological replicates.

To study whether the genes identified by microarray analysis belonged to common pathways, we performed GO analysis on all 143 genes. Only one type of genes was overrepresented in the list namely, immune related genes. In particular, the category formed by IFN inducible genes showed a significant overrepresentation (p-value 6.3E-10). Seven of total possible 37 genes (reported in the database DAVID) identified in this category were altered (p-value  = 8.18E-8). The 7 genes are listed in Table 1. Manual inspection of the list of altered genes revealed one additional IFN induced gene, IFIH1 that is not annotated in the DAVID database (Table 1). Moreover, Ingenuity analysis demonstrated that these 8 genes, as well as many other genes affected by siQKI-7 silencing (Table S1), were included in two major overlapping pathways named “cell-mediated immune response” (score 30 in the ingenuity analysis) and “cellular movement” (score 32). These pathways are illustrated in Supplementary Figure S1 (Fig. S1). None of these genes were affected in their expression levels after silencing of QKI in an oligodendroglial cell line (Table S2). All eight IFN-related genes were down-regulated after silencing of QKI-7. These results indicate a very specific function for QKI-7 and suggest that this transcript may regulate IFN related genes in astrocytes in vivo. The changed expression of these eight genes was further validated by quantitative PCR, that confirmed down-regulation for all eight genes after QKI-7 silencing (Fig. 2).

**Table 1:** GO Classification.

Gene Symbol	Fold Changes			Bipolar a	Schizo b	QRE c	QRE d
	QKI-tot	QKI-5	QKI-7			Core site	Core + half site
IFIT1	1.0	−1.2	−**5.3**	−1	−1	2	2
IFIT2	1.0	1.3	−**4.3**	−1	−1	1	1
MX2	1.1	−1.0	−**3.5**	−1	−1	1	0
MX1	1.0	1.1	−**2.6**	0	−1	1	0
G1P2	1.2	−1.1	−**3.1**	−1	−1	1	1
G1P3	1.3	1.0	−**2.9**	0	0	1	0
GBP1	−1.1	1.1	−**2.3**	−1	0	1	0
IFIH1	−1.2	−1.1	−**2.3**	0	−1	1	1

All 143 genes identified in microarray analysis were also submitted for GO classification as described in the Methods. The table includes expression results for the seven genes included in the most significant GO category, interferon induction (p = 8.18E-8) and one more interferon related gene, IFIH1, not annotated in the DAVID database.

a Expression changes previously reported in postmortem brains from bipolar patients.

b Expression changes previously reported in postmortem brains from patients with schizophrenia.

Data for the columns “Bipolar” and “Schizo” was extracted from the database of Stanley brain bank (http://www.stanleyresearch.org/brain/): “−1”, decreased mRNA expression (p value < 0.05); “1”, increased mRNA expression (p value <0.05); “0”, no significant changes.

c Number of core sites contained within the transcript of the genes included in the table.

d Number of “complete QRE” contained within the transcript of the genes included in the table.

**Figure 3:**
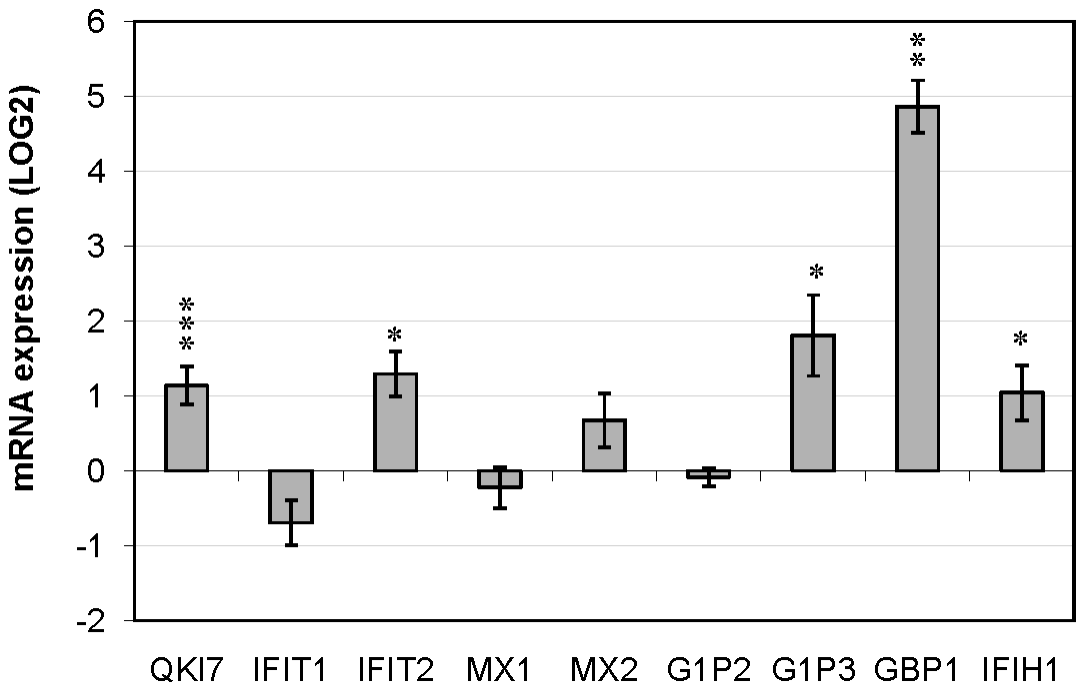
Increased mRNA Expression of Interferon-inducible genes in haloperidol treated U343 cells. Messenger RNA expression levels of IFIT1, IFIT2, MX1, MX2, G1P2, G1P3, GBP1 and IFIH1, relative to reference gene expression (ACTB and GAPDH) were measured after treatment of U343 cells with 0.2 uM haloperidol. As a control for the efficiency of induction, the levels of QKI-7 were also measured and shown to be increased. The level in untreated cells is defined as zero. Asterisk (*) indicates significant deviation in the mRNA levels compared with the untreated cells (p-value <0.05, <0.01, and <0.001 for one, two, and three asterisks, respectively). Mean and standard errors are given based on three biological replicates.

We have recently shown that haloperidol treatment specifically increases QKI-7 in U343 cells [24]. In that study, we did not see an effect of haloperidol in QKI-5 or QKI-6. In other words, the haloperidol effect was splice variant specific [24]. Therefore, we decided to investigate whether haloperidol treatment could reverse the effect of QKI-7 silencing. Fig. 3 shows Real-Time PCR analysis that demonstrates that haloperidol treatment partially reversed the effect of QKI-7 silencing, since several IFN induced genes were significantly increased in a coordinated way with QKI-7 increase (Fig. 3).

### QKI Response Element Analysis

To evaluate whether the genes with altered expression after QKI silencing may be directly regulated due to QKI proteins binding to them, the sequences of the complete set of 143 genes were scanned for the presence of potential QRE binding sites as described in the methods, and reported previously [4]. We first performed a very stringent search for complete QREs, consisting of at least a core site and a half site [4] and we found a significant overrepresentation of these sites among genes altered by QKI silencing compared with a random set of genes present on the arrays (p<0.01, genes with complete QREs are indicated in Table S1). In fact, four of the eight IFN-related genes down-regulated by QKI-7 silencing contained at least one complete QRE (Table 1). Then, we performed a more relaxed search for the presence of QRE core sites without half sites [4]. In this case, many more of the 143 genes were found to include potential QRE sites. For example, all eight genes included in Table 1 contained at least one core-binding site for QKI. Therefore, QKI proteins may directly bind to all eight IFN-related genes.

### Cell viability and morphology

**Figure 4:**
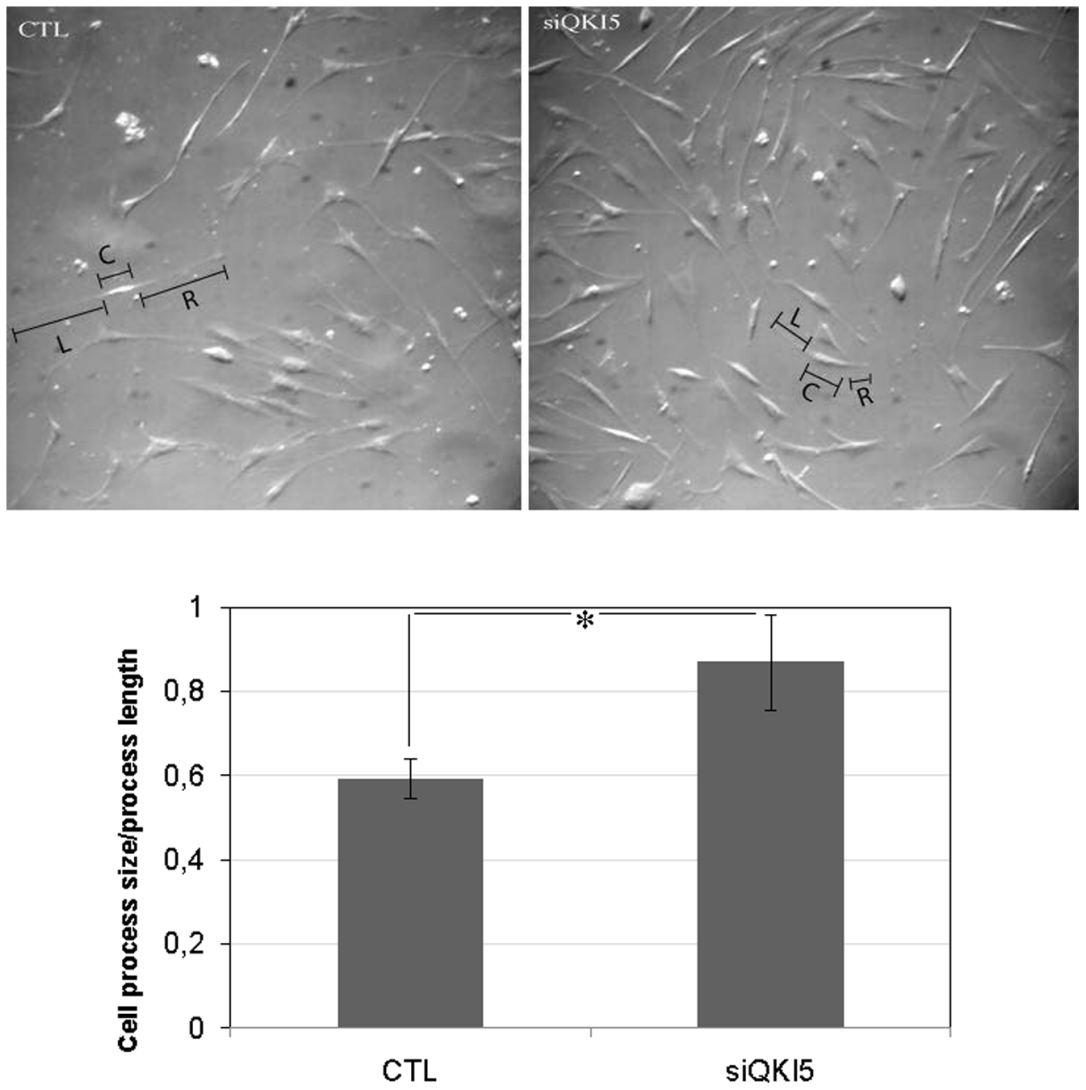
Silencing of QKI-5 changes cell process length. The figure shows the cell process length in untreated (left) and siQKI-5silenced cells (right) after eight days of treatment as described in the methods. The graph below the pictures shows the ratio between the cell body size and cell process length in the untreated and treated cells calculated as described in the methods (p-value <0.05). L indicates the left process, R indicates the right process and C indicates the cell body.

We analysed cell viability and morphology after QKI silencing as described in the methods. No differences in viability were observed after any of the siRNA treatments. Changes in morphology were not observed after one day of treatment with any of the siRNA cocktails. On the other hand, after eight days of treatment, we detected a significantly increased ratio (p-value 0.02) between cell body size and process length in QKI-5 silenced U343 cells (Fig. 4). Changes in ratio were a result of shorter cell process length rather than larger cell bodies. In cells where QKI-7 was silenced, no changes in the ratio between the cell body and process length were detected after eight days of treatment.

## Discussion

We found that siRNA-mediated reduction of QKI-7 resulted in a dramatic down-regulation of well-known IFN induced genes in an astrocytic cell line. This effect was not observed when the cells were silenced with siQKI-tot. These results may seem counterintuitive at first. In fact, siQKI-tot treatment resulted in a dramatic decrease in expression of QKI-7 amongst the other QKI types. However, the microarray results do not show any IFN mRNA targets to be affected by siQKI-tot. These results are not necessarily contradictory. In fact, current research suggests that the balance or proportion between splice variants (rather than changes in each splice variant per se) is important for tissue-specific functions of genes. For example, in the brain, different splice variant compositions can result in different gene function in neuronal tissues [25]. This is also true for QKI function. Indeed, as previously shown by others [26] and by us [6], it is the relationship between the splice variants that is of importance for QKI function. The challenge for the future will be to understand the specific functions of each splice variant and the effect of their proportions, on other cell types in which QKI is expressed.

Among the IFN induced genes, **IFIT1** and **IFIT2** (also named ISG-54 and ISG-56), are known to be highly responsive to viral infection in CNS [27]. **Mx1** and **Mx2** are IFN induced resistance factors produced by neurons, astrocytes and oligodendrocytes [28] and they are critical components of innate immunity against a wide range of RNA virus [29]. **G1P2** (or ISG-15) is rapidly up-regulated after intra-cerebral viral infection [30]. **G1P3** (or IFI-6) is induced by measles virus infection [31]. **GBP1**, or IFN-gamma-inducible human guanylate binding protein 1, belongs to the group of large GTP-binding proteins such as Mx [32]. GBP1 is a key to the protective immunity against microbial and viral pathogens and it is increased in the cerebrospinal fluid of patients with bacterial meningitis [33]. **IFIH1**, or melanoma differentiation associated gene-5 (MDA5), is a pathogen recognition receptor which can, by interacting with RNA virus, initiate antiviral innate immunity and activate genes that encode type I IFNs [34]. Therefore, the coordinated reduction of all of these genes after QKI-7 silencing suggests a regulatory effect of this splice variant in the IFN pathway in astrocytes. In addition, we found that increased QKI-7 expression, induced by an antipsychotic agent, partially reversed the effect of QKI-7 silencing, indicating that QKI-7 levels and IFN-induced gene levels are co-regulated. Interestingly, in a previous study performed in our group, no effect on QKI-5 and QKI-6 was observed in treated U343 cells. In addition, antipsychotic agents did not have an effect of QKI-7 in oligodendrocytes. The results indicate that the effect of haloperidol on QKI-7 is splice variant and cell type specific. All combined results suggest a novel role for QKI-7 in astrocytes as a specific regulator of IFN-related pathway.

Astrocytes, the most abundant glial cell population, are important for brain homeostasis and neuronal function [35]. They display an array of receptors involved in innate immunity and have the ability to secrete soluble mediators which have an impact on both innate and adaptive immune responses [36]. Because of the plethora of roles to maintain central nervous system (CNS) functions, it is not surprising that dysfunctional astrocytes are becoming recognized as key players in many CNS disorders [22]. Among these diseases, schizophrenia may not be an exception. In fact, we have previously shown specific down-regulation of QKI-7 in the prefrontal cortex (PFC) of schizophrenia patients [6]. In this previous work, it was not possible to analyse the specific cell type responsible for QKI-7 down-regulation. On the other hand, it has previously been suggested that alterations in schizophrenia patients occur both in oligodendrocyte- and astrocyte-related genes [37], [38]. Therefore, it is very likely that QKI down-regulation has important physiological consequences in both oligodendrocytes and astrocytes. All of the results discussed here can be summarized in a testable hypothesis related to a novel function of a specific QKI splice variant in astrocytes. According to this hypothesis, expression of splice variant QKI-7 regulates IFN induced gene expression in astrocytes. When QKI-7 transcript is decreased, the innate immune function of astrocytes is modified, making the CNS more vulnerable to environmental insults and possibly increasing the risk for mental disorders such as schizophrenia. This hypothesis is well in line with previous reports discussing inflammatory components in schizophrenia [39], [40], [41], [42], [43].

## Materials and Methods

### Cell Cultures

The human astrocytoma (U343) cell line was originally established by Professor Bengt Westermark's group [44]. The human oligodendroglial cell line (HOG) was established from a surgically removed human oligodendroglioma [23] and they were kindly provided to us by Dr Campagnoni and Dr De Vellis. The cells were cultured on six-well tissue culture plates (NUNC) in monolayer, using Dulbecco's Modified Eagle Medium (DMEM) (Invitrogen, Sweden) supplemented with 5% fetal calf serum (FCS) (Invitrogen, Sweden) and 1% penicillin-streptomycin solution (PEST, Invitrogen, Sweden) at 37°C and 5% CO_2_.

### RNA silencing

A pool of four siRNA duplexes was designed to silence all QKI splice variants (QKI-tot), while two other siRNA pools were designed to specifically silence splice variants QKI-5 or QKI-7 (DHARMACON, Custom SMARTpool® siRNA Design). All siRNA sequences are shown in Table 2, and the positions of the sequences with respect to the QKI gene structure are shown in Fig. 1A. siRNA for GAPDH (Ambion Silencer™ GAPDH siRNA) was used as a positive control for transfection. Also as a control for the specificity of siRNA silencing, GAPDH silencing did not affect expression of QKI. U343 cells were split to new six-well tissue culture plates (NUNC) one or two days before transfection. Transfection mix consisted of 2 µl siRNA (50 µM), 10 µl lipofectamine2000 (Invitrogen) and 588 µl OptiMEM (Invitrogen), with a final volume of 500 µl. The negative control (transfection mix without siRNA) contained 10 µl lipofectamine2000 and 490 µl OptiMEM. The transfection and control mix were added to cell suspensions (10^6^ cells/ml) and the cell cultures were incubated for 24 hours. Biological triplicates were prepared for the siRNA experiment as well as the negative controls. After 24 hours the medium was replaced with 1000 µl fresh DMEM (without FCS and PEST) and transfected and control cells were incubated for additional 24 hours (or more for viability and morphology studies). The DMEM was removed from the wells. 1000 µl PBS was added and the cells were resuspended, moved to eppendorph tubes and centrifuged (2500 rpm for 5 minutes). The pellets were resuspended an additional time in 1000 µl PBS and centrifuged. The supernatant was removed and the pellet was resuspended in 500 µl Trizol Reagent (Invitrogen) and stored at −20°C until RNA extraction.

**Table 2:** Sequences of duplexes designed for three siRNA cocktails used to silence all QKI splice variants (siQKI-tot) or specifically splice variant 5 (siQKI-5) or 7 (siQKI-7).

siRNA coctail	siRNA sequences
siQKI-tot	GAACAGAGCAGAAAUCAAAUU GCCCGAAGCUGGUUUAAUCUU GGGAGCAUCUAAAUGAAGAUU CCAGCUGGCCCUACCAUAAUU
siQKI-5	GACAGAUGGUCCUUAACUAUU CACGUUACCUUGAUGCAAAUU GCACUUGUCCGUUCGUCUUUU GCCUACAUCUAAACACUUGUU
siQKI-7	GCAGACAUGUGUGUUGGUAUU GUGAGGAGAUUGGUAUUAGUU ACACUAAGAUUUAAACUCGUU AAUAAUAAGUGCCCAAUGAUU

### RNA Extraction

100 µl Chloroform was added to each of the cell samples resuspended in trizol. All samples were mixed gently for 15 seconds, incubated for 2–3 minutes at room temperature and centrifuged at 12000 g for 15 minutes. The aqueous phase, containing RNA, was collected and 250 µl isopropanol was added. The tubes were incubated for 10 minutes at room temperature and then centrifuged at 12000 g for 10 minutes. The supernatant was removed and the pellets were washed gently in 70% ethanol (−20°C). The samples were centrifuged at 7500 g for 5 minutes. The ethanol was removed and the pellets were dried for 10 minutes. The RNA was dissolved in 10 µl RNase-free water. The samples were quality controlled and quantified on a NanoDrop® ND-1000, and stored at −70°C until use.

### Microarray hybridizations

About 10 µg of total RNA from each biological replicate were used for a reverse transcription reaction with the Genisphere® Labelling and Detection Kit, 3DNA Array 900 Cy3/Cy5. The cDNAs produced from each silencing experiment were mixed with cDNAs from control cells and the mix was hybridized to slides printed with 46 k human cDNA clones (Microarray Resource Centre, Royal Institute of Technology, Sweden). Each experiment included three biological replicates and dye swaps. Detailed information on the experimental design is available on ArrayExpress (accession number E-MEXP-1393).

### Microarray Analysis

Microarrays were scanned at 10 µm resolution using a GenePix4100A scanner (Axon Instruments, Inc.). Spots on the resulting images were quantified with the software package GenePix Pro 5.1 (Axon Instruments, Inc.). The mean intensity of the two samples (Cy5-labelled sample  = R, and Cy3-labelled sampled  = G) were used to calculate the log-transformed ratio between the two samples for each spot: M = log2 (R/G). The slide images were manually inspected and faulty spots as well as whole faulty sections were removed from the analysis. The resulting data was exported and the SAS statistical system was used to normalize the slides for spatial trends (different signal intensities in different parts of the arrays) and for red-green bias (different labeling intensities for the two dyes). To remove spatial trends, the loess procedure was used in both x and y direction on the arrays. Spots with a mean intensity below their local background were excluded and the normalized M value for missing values was set to zero. Using the GLM procedure in SAS a statistical model was created. In this model, the normalized R-value was the dependent variable explained by one of the following siRNA treatments: ‘siQKI-tot’ (U343 cells treated with siRNA targeting QKI-total), ‘siQKI-5’ (U343 cells treated with siRNA targeting QKI-5) or ‘siQKI-7’ (U343 cells treated with siRNA targeting QKI-7). Penalized F was used to identify differentially expressed genes. Permutation tests (n = 5000), in which the ID of the arrays was randomized, were used to find appropriate cut off values for significance.

### Gene Categories and gene networks

Using DAVID online tools, we tested overrepresentation of gene ontology (GO) categories in the different lists of differentially expressed genes after each siRNA treatment. The program delivered a list of overrepresented categories as well as their test statistics [45]. We studied whether QKI silencing modified networks of related genes using ingenuity pathway analysis (http://www.ingenuity.com/).

### QRE Search

Differentially expressed genes were searched for the presence of potential QREs in their sequences using TFBS module [46]. QRE is a bipartite binding site separated by less than 20 base pairs. Two QRE matrixes corresponding to each QRE half site obtained by in vitro SELEX experiments [4] were transformed to a single pattern weighted matrix used to perform a search for potential sites using a Perl script. After potential core sites were identified, neighboring sequences both upstream and downstream of these potential sites were searched for the presence of a potential half binding sites using a second weighted matrix. Only genes containing both a potential core binding site and a potential half binding site were considered as positive for the presence of a “complete QRE”. To test for over-representation of complete QREs in genes affected by silencing of QKI, Fisher's exact test was performed.

### Real-Time PCR Analysis

From each biological replicate from the silencing experiments, 500 ng of total RNA was reverse transcribed to cDNA using TaqMan RT reagents (Applied Biosystems, New Jersey). The final concentrations of reagents were: 1 x TaqMan RT buffer, 5.5 mM MgCl2, 2 mM dNTP mixture, 2.5 µM Oligo(dT) primers, 0.4 U/µl RNase inhibitor and 1.25 U/µl MultiScribe reverse transcriptase in RNase-free water to a total volume of 25 µl. The reaction mix was incubated at 25°C for 10 minutes (primer annealing), 48°C for 1 hour (synthesis) and 95°C for 5 minutes (enzyme inactivation). The resulting cDNA samples were stored at −20°C. All real-time PCR experiments were performed on an ABI Prism 7000 Sequence Detector System (Applied Biosystems, Foster City, USA) using 96-well plates (ABI). The TaqMan reaction mix included 9.2 µl nuclease-free H_2_O, 9.8 µl TaqMan ® Universal PCR Master Mix (ABI), 0.66 µl forward primer (10 µM), 0.66 µl reverse primer (10 µM) and 0.66 µl probe (5 µM) per sample. The primers and probes for ACTB, GAPDH and different QKI splice variants were described before [6]. The expression of IFIT1, IFIT2, MX1, MX2, G1P2, G1P3, GBP1 and IFIH1 was measured using SYBR green. Primer sequences are summarized in Table 3. The SYBR green reactions contained 9.5 µl nuclease-free H_2_O, 10 µl Power SYBR ® Universal PCR Master Mix(ABI), 0.75 µl forward primer (10 µM), and 0.75 µl reverse primer (10 µM). All reactions included 4 µl of cDNA and RNase-free water to a total volume of 25 µl. The Real-Time PCR was performed as follows: 50°C for 2 minutes (UNG incubation) and 95°C for 10 minutes (AmpliTaq Gold activation), followed by 40 cycles of 95°C for 10 seconds and 60°C for 1 minute. A dissociation step was added for SYBR green runs. For each sample, gene expression was quantified using a standard curve and normalized against the expression of the endogenous control genes GAPDH and ACTB. In addition, the gene expression was normalized to a calibrator sample (untreated control cultures) to estimate the change in gene expression after silencing. To statistically evaluate the significance of the expression differences two-tailed t-tests were used.

**Table 3:** Primer sequences used for RT-PCR.

Gene	5′-Forward Primers-3′	5′-Reverse Primers-3′
IFIT1	CAGAACGGCTGCCTAATTTACA	CAGACTATCCTTGACCTGATGATCA
IFIT2	CAGCTGAGAATTGCACTGCAA	CGTAGGCTGCTCTCCAAGGA
MX1	ATTTCGGATGCTTCAGAGGTAGA	CCCGGCGATGGCATT
MX2	CAGCCACCACCAGGAAACA	TTCTGCTCGTACTGGCTGTACAG
G1P2	TGGCGGGCAACGAATT	GGGTGATCTGCGCCTTCA
G1P3	CCTCAAGTGATCCTCCTGTCTCA	TCGTCGGCGCATGCTT
GBP1	CCAGTTGCTGAAAGAGCAAGAGA	TCCCTCTTTTAGTAGTTGCTCCTGTT
IFIH1	GACTATCAAATAAATGGTGAAATCATCTG	CCTTTGTGCACCATCATTGTTC

### Cell viability and cell morphology

To investigate the effect that different splice variants have on cell viability and morphology, cell cultures were silenced with siRNA targeting QKI-5 and QKI-7, as described above. Cell viability was measured using a MTT reduction assay as described [47]. The cell body size and length of cell processes were measured in untreated and siRNA treated cells, after one and eight days of treatment.

## Supporting Information

Figure S1 Ingenuity analysis of gene networks affected by silencing of QKI-7. Ingenuity analysis (http://www.ingenuity.com/) was performed to search for networks of genes affected by QKI-7 silencing. “Cell-mediated immune response” is illustrated on the left part of the figure (score 30) and “Cellular movement” in the right part (score 32). Genes up-regulated by QKI-7 silencing are marked in red and genes down-regulated by the same treatment are marked in green. Genes not found to be affected by QKI-7 silencing are marked in white. The microarray results obtained for the genes marked in green and red are shown in Supplementary Table 1, part C, where these genes are marked with the same color. The lines connecting genes represent direct interaction including transcriptional regulation and protein-protein interaction between them. The shapes enclosing gene names represent different types of factors, such as flat circles for transcription factors.
(0.07 MB PDF)

Table S1 Three groups of genes changed in response to silencing of QKI splice variants in U343 cells. The table includes microarray expression results for genes with the most significantly modified mRNA expression levels after silencing experiments. Cut-off for significance was calculated as described in the methods. Part A. Genes altered after silencing with siQKI-tot, a cocktail that targets all splicing variants. Part B. Expression changes after silencing with siQKI-5. Part C. Expression changes after silencing with si-QKI-7. Genes marked with color correspond to the same genes included in the two pathways found after the ingenuity analysis (Fig. S1). Fold changes are given in log2 scale a Expression changes previously reported in postmortem brains from bipolar patients. b Expression changes previously reported in postmortem brains from patients with schizophrenia. Data for the columns “Bipolar” and “Schizo” was extracted from the database of the Stanley brain bank (http://www.stanleyresearch.org/brain/): “−1”, decreased mRNA expression (p value <0.05); “1”, increased mRNA expression (p value <0.05); “0”, no significant changes. c Number of potential QKI response elements (QRE) contained within the transcript of the genes included in the table calculated as described in the methods.
(0.07 MB PDF)

Table S2 Expression of IFN-related genes in two cell lines after QKI-7 silencing. The table shows that all 8 IFN-related genes were down-regulated after QKI-7 silencing of U343 cells while their expression was not significantly affected in HOG cells. Asterisk (*) indicates significant deviation in the mRNA levels compared with the cells that were not treated with siQKI-7 (p-value <0.05, <0.01, and <0.001 for one, two, and three asterisks, respectively).
(0.01 MB DOCX)
