# Supplementary figures and images for: QKI-7 Regulates Expression of Interferon-Related Genes in Human Astrocyte Glioma Cells
Source: PLoS One. 2010 Sep 29;5(9):e13079. doi: 10.1371/journal.pone.0013079 (PMC2947523; doi:10.1371/journal.pone.0013079)

© 2000-2009 Ingenuity Systems, Inc. All rights reserved.

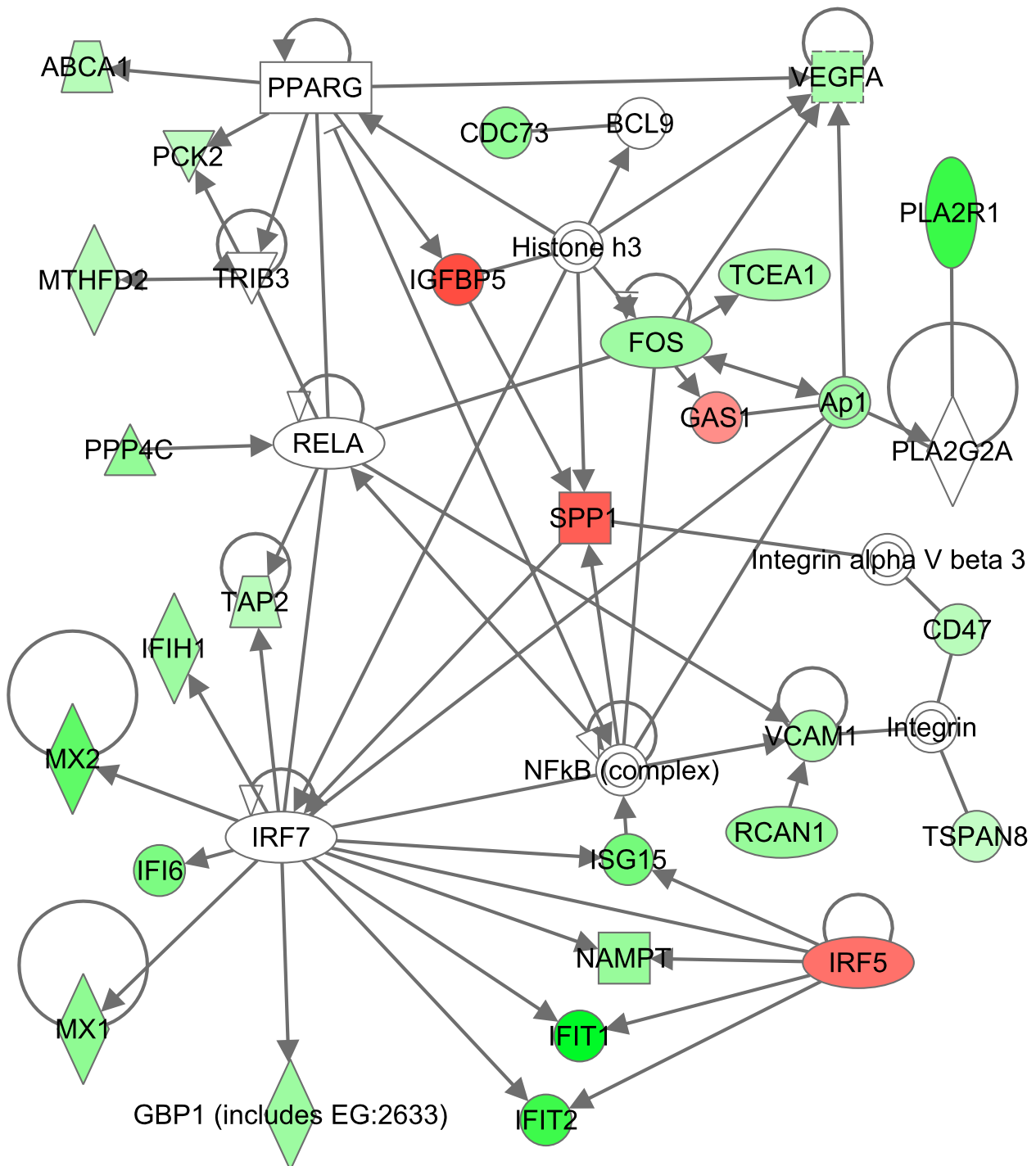

Supplement: Figure S1 — Ingenuity analysis of gene networks affected by silencing of QKI-7. Ingenuity analysis (http://www.ingenuity.com/) was performed to search for networks of genes affected by QKI-7 silencing. “Cell-mediated immune response” is illustrated on the left part of the figure (score 30) and “Cellular movement” in the right part (score 32). Genes up-regulated by QKI-7 silencing are marked in red and genes down-regulated by the same treatment are marked in green. Genes not found to be affected by QKI-7 silencing are marked in white. The microarray results obtained for the genes marked in green and red are shown in Supplementary Table 1, part C, where these genes are marked with the same color. The lines connecting genes represent direct interaction including transcriptional regulation and protein-protein interaction between them. The shapes enclosing gene names represent different types of factors, such as flat circles for transcription factors. (0.07 MB PDF) [file pone.0013079.s001.pdf]
