# Supplementary material for: QKI-7 Regulates Expression of Interferon-Related Genes in Human Astrocyte Glioma Cells
Source: PLoS One. 2010 Sep 29;5(9):e13079. doi: 10.1371/journal.pone.0013079 (PMC2947523; doi:10.1371/journal.pone.0013079)
Supplement: Table S1 — Three groups of genes changed in response to silencing of QKI splice variants in U343 cells. The table includes microarray expression results for genes with the most significantly modified mRNA expression levels after silencing experiments. Cut-off for significance was calculated as described in the methods. Part A. Genes altered after silencing with siQKI-tot, a cocktail that targets all splicing variants. Part B. Expression changes after silencing with siQKI-5. Part C. Expression changes after silencing with si-QKI-7. Genes marked with color correspond to the same genes included in the two pathways found after the ingenuity analysis (Fig. S1). Fold changes are given in log2 scale a Expression changes previously reported in postmortem brains from bipolar patients. b Expression changes previously reported in postmortem brains from patients with schizophrenia. Data for the columns “Bipolar” and “Schizo” was extracted from the database of the Stanley brain bank (http://www.stanleyresearch.org/brain/): “−1”, decreased mRNA expression (p value <0.05); “1”, increased mRNA expression (p value <0.05); “0”, no significant changes. c Number of potential QKI response elements (QRE) contained within the transcript of the genes included in the table calculated as described in the methods. (0.07 MB PDF) [file pone.0013079.s002.pdf]

PART A siQKItot

Gene Symbol	Fold Change (LOG 2)	Bipolar [a]	Schizo [b]	QRE [c]	CLONE ID
QKI	**-2,2**	-1	1	6	139167
STMN3	**1,6**	-1	0	0	1631253
SAT	**-1,1**	-1	0	0	359835
RGS4	**1,7**	-1	-1	0	22355
IGFBP5	**-1,3**	0	0	0	45542
EFEMP1	**0,9**	0	1	3	1492230
SAT	**-1,2**	-1	0	0	897864
AKR1C2	**-1,2**	-1	-1	6	2449395
CDW92	**-0,9**	-1	0	3	838689
PLCB4	**0,9**	0	0	1	190325
KIAA0101	**0,8**	0	0	2	342640
ChGn	**-0,8**	0	1	1	1759290
TNFRSF11B	**0,8**	0	0	2	665356
DYRK2	**0,8**	0	0	0	138737
CYR61	**0,8**	0	0	1	360254
AF1Q	**0,9**	-1	0	1	812105
SCG2	**0,9**	-1	-1	1	174627
TNPO1	**-1,1**	0	0	5	701371
FST	**0,8**	0	0	0	434768
AGT	**0,9**	1	1	0	2019101
PDE4D	**-0,7**	0	0	3	746321
LYPDC1	**0,9**	0	0	0	755612
C21orf7	**0,9**	0	0	3	826072
MAFB	**-0,8**	-1	-1	2	77193
Transcribed	**-0,7**			3	41463
AKR1C1	**-0,8**	-1	-1	0	199648
CDC40	**-0,8**	0	0	0	856639
JAG1	**-0,8**	0	0	4	141815
ATP8B3	**-0,8**	0	0	0	2450261
KIF5C	**0,6**	0	0	1	278430
DDR1	**-1,0**	-1	1	0	841384
PLCB4	**1,0**	0	0	1	51916
S100A7	**-0,8**	0	0	0	1088345
WDR9	**0,7**	0	1	5	814975
OLFML2B	**-0,8**	-1	0	0	265694
DNER	**0,7**	0	0	0	281392

PART B siQKI-5

Gene Symbol	Fold Change (LOG 2)	Bipolar [a]	Schizo [b]	QRE [c]	CLONE ID
CCNL2	**-1,1**	-1	1	0	506623
CREG1	**-1,0**	-1	0	0	85409
CHST2	**1,2**	-1	-1	0	431301
PRDX3	**-1,0**	-1	0	0	50888
CALU	**-1,2**	-1	0	2	144881
TDO2	**0,9**	0	1	0	461535
STMN3	**1,1**	-1	0	0	1631253
QKI	**-1,3**	-1	1	6	139167
PIAS1	**0,8**	0	1	1	32565
AGPAT3	**-0,9**	0	0	3	562067
RPN1	**-0,9**	-1	0	0	137189
SCD	**-1,0**	-1	-1	1	810711
EPAS1	**-0,8**	0	0	3	131979
CXADR	**0,8**	-1	0	0	276712
RAB6A	**-1,0**	-1	-1	1	172440
Transcribed	**0,8**	0	0	0	244879
TMSB4Y	**-0,7**	0	0	1	280758
ACLY	**-0,9**	-1	-1	4	502622
Transcribed	**-0,7**	-1	0	0	53315
TFB1M	**-0,8**	-1	0	0	788317
MGC35033	**0,7**	0	0	1	745487
EIF4A2	**0,9**	-1	0	1	1636539
Transcribed	**-0,7**			1	277634
COMT	**-0,8**	0	0	0	1635260
LAMP2	**-0,9**	-1	1	0	289615
BRPF1	**-0,8**	0	0	1	121776

PART C siQKI-7

Gene Symbol	Fold Change (LOG 2)	Bipolar [a]	Schizo [b]	QRE [c]	CLONE ID
ABCA1	**-0,9**	0	0	3	827168
BST2	**-1,9**	-1	0	0	811024
FAM129A	**-0,8**	0	0	0	399331
C20orf112	**-0,7**	0	0	0	841146
PPDPF	**1,0**	0	0	0	1606275
C5orf13	**-0,7**	-1	-1	1	241432
SLC22A23	**-0,9**	0	0	2	295939
CAST	**0,8**	-1	0	4	591381
CD47	**-0,9**	-1	0	2	813552
CDK5R2	**-0,8**	0	-1	0	1751068
CG14903-PA	**0,8**			1	1469629
CHST2	**1,2**	-1	-1	0	431301
CYR61	**-0,7**	0	0	1	360254
DKFZp434O	**-0,8**	0	0	0	1842518
RCAN1	**-1,2**	-1	0	1	2556526
FLJ21103	**-0,9**	0	0	4	884388
FOS	**-1,2**	0	-1	0	811015
G1P2=ISG15	**-1,6**	-1	-1	1	742132
G1P3=IFI6	**-1,5**	0	0	0	782513
GAS1	**1,0**	0	0	0	365826
GBP1	**-1,2**	-1	0	1	712292
HIPK2	**-1,1**	0	0	1	950603
HLA-DPA1	**0,9**	-1	-1	5	868332
CDC73	**-1,3**	-1	0	1	784177
ID3	**1,4**	0	0	0	756405
IFIH1	**-1,2**	0	-1	1	1520357
IFIT1	**-2,4**	-1	-1	2	2550047
IFIT2	**-2,1**	-1	-1	1	1527063
IGFBP5	**1,6**	0	0	0	45542
IGLL1	**1,3**	0	0	0	344134
IRF5	**1,3**	0	0	0	260035
JUB	**-0,9**	-1	0	0	812974
KCNG1	**-1,2**	0	0	0	2321529
KIF5C	**0,7**	0	0	1	278430
LRRC17	**1,2**	0	-1	0	2010012
MATN2	**0,8**	0	0	0	366100
MGC3265	**0,8**	0	1	1	435447
MGC39900	**0,8**	0	0	0	306771
MTHFD2	**-0,9**	0	0	1	2014034
MX1	**-1,4**	0	-1	0	815542
MX2	**-1,8**	-1	-1	0	701481
NALP1	**1,2**	0	0	0	1597813
NEBL	**-1,4**	-1	0	3	796643
OCIAD1	**-0,6**	0	0	0	132569
PAI-RBP1	**-0,8**	-1	-1	1	823692
PARP14	**-1,3**	-1	0	1	66815
NAMPT	**-1,2**	0	0	1	823665
PCK2	**-0,8**	0	0	1	1845146
PLA2R1	**-2,1**	0	0	4	511303
PNMT	**1,3**	0	0	0	289857
PPP1R1A	**1,0**	0	0	0	796268

PART C siQKI-7 continued

Gene Symbol	Fold Change (LOG 2)	Bipolar [a]	Schizo [b]	QRE [c]	CLONE ID
PPP4C	**-1,3**	0	0	0	772455
RGC32	**-0,6**	0	1	0	487287
Similar to ferritin	**1,1**			0	1712407
SLC7A1	**-0,8**	0	0	1	134712
SORBS2	**-0,9**	0	0	0	151240
SPANXA2	**1,1**	0	0	0	1839241
SPP1	**1,4**	-1	0	0	378461
STC2	**-1,3**	0	0	1	130057
STC2	**-0,9**	0	0	1	823578
TAP2	**-0,9**	-1	-1	4	753236
TCEA1	**-1,1**	-1	0	1	163174
TIMP3	**-1,9**	1	1	0	754106
TIMP3	**-1,1**	1	1	0	489519
TM4SF3=TSPAN8	**-0,7**	-1	0	0	509731
TTC7A	**-1,2**	0	0	1	153650
TUBE1	**-0,8**	0	0	2	279460
VCAM1	**-1,0**	0	0	1	44477
VEGF	**-1,1**	-1	-1	4	34778
WDNM1-like	**-0,9**			0	503051
ZDHHC23	**-0,7**	0	0	3	52704
