# Supplementary material for: QKI-7 Regulates Expression of Interferon-Related Genes in Human Astrocyte Glioma Cells
Source: PLoS One. 2010 Sep 29;5(9):e13079. doi: 10.1371/journal.pone.0013079 (PMC2947523; doi:10.1371/journal.pone.0013079)
Supplement: Table S2 — Expression of IFN-related genes in two cell lines after QKI-7 silencing. The table shows that all 8 IFN-related genes were down-regulated after QKI-7 silencing of U343 cells while their expression was not significantly affected in HOG cells. Asterisk (*) indicates significant deviation in the mRNA levels compared with the cells that were not treated with siQKI-7 (p-value <0.05, <0.01, and <0.001 for one, two, and three asterisks, respectively). (0.01 MB DOCX) [file pone.0013079.s003.docx]

|  |  | |
| --- | --- | --- |
| **Gene Symbol** | **Fold Changes**  **(LOG2)** | |
|  |  |  |
|  | HOG QKI-7 | U343 QKI-7 |
|  |  |  |
| IFIT1 | -0,2 | -2,4*** |
| IFIT2 | -0,2 | -2,1** |
| MX2 | -0,2 | -1,8** |
| MX1 | -0,1 | -1,4** |
| G1P2 | 0,1 | -1,6*** |
| G1P3 | 0,0 | -1,5** |
| GBP1 | 0,1 | -1,2* |
| IFIH1 | 0,0 | -1,2* |
|  |  |  |
